# Exogenous selection rather than cytonuclear incompatibilities shapes asymmetrical fitness of reciprocal *Arabidopsis* hybrids

**DOI:** 10.1002/ece3.1474

**Published:** 2015-03-25

**Authors:** Graham Muir, Paola Ruiz-Duarte, Nora Hohmann, Barbara K Mable, Polina Novikova, Roswitha Schmickl, Alessia Guggisberg, Marcus A Koch

**Affiliations:** 1 Centre for Organismal Studies, Department of Biodiversity and Plant Systematics, University of HeidelbergD-69120, Heidelberg, Germany; 2 Institute of Biodiversity, Animal Health and Comparative Medicine, University of GlasgowGlasgow, G12 8QQ, U.K; 3 Gregor Mendel Institute, Austrian Academy of SciencesVienna, Austria; 4 Institute of Integrative Biology, ETH Zürich8092, Zürich, Switzerland

**Keywords:** Asymmetric reproductive isolation, cytonuclear incompatibilities, Darwin's corollary to Haldane's rule, hybrid inviability, postzygotic selection

## Abstract

Reciprocal crosses between species often display an asymmetry in the fitness of F_1_ hybrids. This pattern, referred to as isolation asymmetry or Darwin's corollary to Haldane's rule, is a general feature of reproductive isolation in plants, yet factors determining its magnitude and direction remain unclear. We evaluated reciprocal species crosses between two naturally hybridizing diploid species of *Arabidopsis* to assess the degree of isolation asymmetry at different postmating life stages. We found that pollen from *Arabidopsis arenosa* will usually fertilize ovules from *Arabidopsis lyrata*; the reverse receptivity being less complete. Maternal *A. lyrata* parents set more F_1_ hybrid seed, but germinate at lower frequency, reversing the asymmetry. As predicted by theory, *A. lyrata* (the maternal parent with lower seed viability in crosses) exhibited accelerated chloroplast evolution, indicating that cytonuclear incompatibilities may play a role in reproductive isolation. However, this direction of asymmetrical reproductive isolation is not replicated in natural suture zones, where delayed hybrid breakdown of fertility at later developmental stages, or later-acting selection against *A. arenosa* maternal hybrids (unrelated to hybrid fertility, e.g., substrate adaptation) may be responsible for an excess of *A. lyrata* maternal hybrids. Exogenous selection rather than cytonuclear incompatibilities thus shapes the asymmetrical postmating isolation in nature.

## Introduction

In many cases of hybridization, there is an asymmetry in the fitness of reciprocal F_1_ hybrid crosses (Tiffin et al. 2001; Turelli and Moyle 2007; Bolnick et al. 2008). This asymmetry has been called isolation asymmetry or Darwin's corollary to Haldane's rule (Turelli and Moyle 2007). The pattern cannot be explained by Dobzhansky–Muller incompatibilities (DMIs) between autosomal loci because reciprocal hybrids have the same autosomal genotype (Turelli and Moyle 2007). The nuclear genome is inherited equally from both parents and, aside from interactions between hybrid nuclear genotypes and their environment, is not transmitted differentially. Instead, isolation asymmetry is probably due to DMIs involving uniparentally inherited factors or interactions between the maternal and hybrid progeny's genomes (Turelli and Moyle 2007). These nonnuclear contributions may include cytoplasmic effects (Burton et al. 2013) or genomic imprinting (e.g., unequal contributions to the endosperm; Gehring 2013). Such effects are transmitted differentially (asymmetrically) and thus manifest themselves as fitness differences in reciprocal crosses between species (e.g., Etterson et al. 2007; Martin and Willis 2010; Goodwillie and Ness 2013).

Theory suggests that the direction with the lowest fitness in reciprocal crosses between species (isolation asymmetry) will vary with the relative rates of cytoplasm and nuclear evolution in the parental species (Turelli and Moyle 2007). If these rates differ between parental species, then crosses with the lower rate of offspring viability are those in which the maternal parent originates from the species with a higher comparative rate of cytoplasm evolution (as there is a higher probability of cytonuclear incompatibilities; Bolnick et al. 2008). This theory suggests that the direction of asymmetry might be predictable from the fitness of maternal hybrid species present in natural suture zones, as well as which of the two maternal species accumulates nucleotide substitutions in the cytoplasm at a higher rate.

Here we analyze reciprocal-cross data between two naturally hybridizing species of *Arabidopsis* (*Arabidopsis arenosa* and *Arabidopsis lyrata*) to examine patterns of asymmetry through time at two stages of isolation: seed set and seed germination. These closely related species are self-incompatible hermaphrodites with a sympatric range in parts of central Europe where they hybridize. In these suture zones, both diploid and tetraploid hybrids exhibit *A. lyrata* maternal backgrounds (Schmickl and Koch 2011; M. Koch, N. Hohmann, G. Muir, unpubl. data), suggesting the presence of isolation asymmetry in fertility (or survivorship) of reciprocal hybrid crosses.

The results of these crosses support the notion that postmating barriers are generally strong and contribute significantly to asymmetrical reproductive isolation in these two species, while never solely leading to complete isolation (for a STRUCTURE analysis, Pritchard et al. 2000; of gene pools and admixture between these two diploids, see Hohmann et al. 2014). Given the split time of these two lineages, based on fossil-calibrated divergence time estimates (1–2 Myr, Hohmann et al. unpubl. data), the potential for accumulating reproductive barriers during speciation has been limited. Together with the direction of asymmetry in the wild, we discuss the factors that shape the evolution and fitness of interspecific *Arabidopsis* hybrids.

## Materials and Methods

### Source material, artificial crosses, and experimental design

We conducted reciprocal crosses in the greenhouse between a diploid member of the *Arabidopsis arenosa group* (*A. carpatica*, hereafter *A. arenosa*) and *A. lyrata* subsp. *p*e*traea* (hereafter *A. lyrata*). Material for the crosses was raised from open-pollinated seeds collected in Nízke Tatry and Vel'ká Fatra, central Slovakia (*A. arenosa*), and from the foothills of the eastern Austrian limestone Forealps (*A. lyrata*). Eight reciprocal crosses (between couples) were made between these two species; each couple producing full-sib offspring. A further six and four (half-sib) conspecific crosses were conducted within *A. arenosa* and *A. lyrata*, respectively, to control for differences in the receptivity or fecundity of the parental taxa. Ten to fifteen pollinations were made by hand for each parental cross. All pollinations were performed in a pollinator-free environment and conducted without competition; pollen from only a single paternal parent was placed on each stigma. Measures for crossing success were seed set and the proportion of seeds that were viable (F_1_ seed viability). Seed viability was measured for >20 seeds for each cross performed and assessed by germination ability (% germination of seeds sown).

Prior to germination, seeds were washed three times for 10 min in a 10% sodium hypochlorite solution and washed thoroughly in sterile water. After partially drying, seeds were plated on agar plates containing half-strength salts and vitamins, 1.5% sucrose, and 0.8% agar (Murashige and Skoog 1962). The plates were placed for 2 days at 4°C, and then seedlings were planted in medium containing a 3:1 mixture of a peat-based compost and 1–3 mm grit. Potted seedlings were raised under short-day conditions (8 h of light/16 h of dark) at 22°C.

### Seed traits, fitness, and statistical analysis

Reproductive isolation was defined separately for each fitness-related parameter: seed set and F_1_ seed viability. Fitness was measured as the total number of fully mature seeds produced per maternal plant for each cross performed, assessed over an extended 5-month period (May–September).

Likelihood ratio chi-square tests were used to test whether the success of a cross was significantly affected by which species was the pollen parent and which species was the seed parent. Separate tests were conducted for each of the stages at which isolation was measured.

The following morphological traits for each cross and their parents were measured from a sample of five siliques (seeds included) per individual: silique length, seed width and length (to the nearest 0.1 *μ*m), and ratios of seed and wing size (minimum and maximum length ÷ width). Images of these traits were analyzed with WinFolia image analysis software (Regent Instrument Inc., Quebec, Canada). A principal component analysis was conducted on these five values in SPSS Statistics for Windows v19.0 (IBM, Armonk, NY) to identify key components of the fruiting structure that explained the greatest possible variance in the data, and to group and/or separate parents/F_1_ progeny visually.

## Results

### Hybrid fitness depends on the direction of the cross

**Figure 1:**
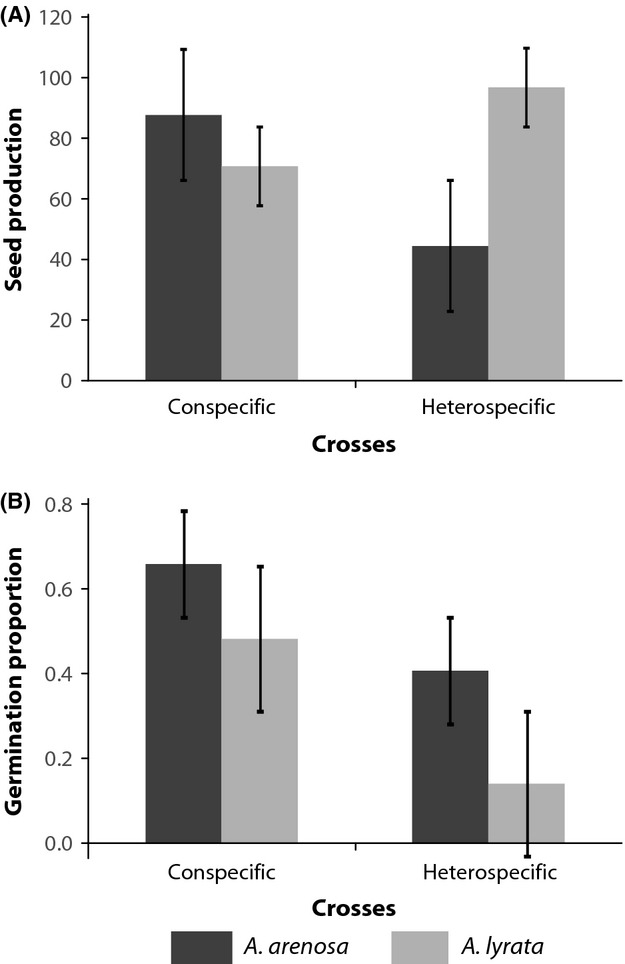
Relative mean fitness of F_1_ individuals from intra- and interspecific experimental pollinations. Seed production (A) was defined as the total number of seeds collected from viable siliques for each cross performed. Germination (B) was defined by the number of germinating seeds per 100 seeds sown. Maternal parents are grouped by species. For each parental cross (intraspecific/interspecific), four to eight F_1_ families (replicates), respectively, were generated.

Significant asymmetries in the strength of reproductive isolation between *A. arenosa* and *A. lyrata* were found at both stages of isolation (Fig.1A and B). All eight heterospecific crosses performed to generate the hybrid F_1_ generation were successful in at least one direction. However, the success of the crosses was dependent on the species of the maternal and the paternal parent, that is, the direction of the cross affected either seed production or germination and thus success rates. For crosses between *A. lyrata* (as the maternal parent, *♀*)* *× *A. arenosa* (as the pollen donor, ♂), seeds were produced in higher quantities than the reciprocal cross (Fig.1A), with a twofold reduction when *A. arenosa* was the maternal parent. *A. lyrata* maternal parents produce on average almost twice as many seeds as *A. arenosa* maternal parents (Fig.1A; Mann–Whitney *U*-test, *P* = 0.029).

The asymmetry was prevalent in all eight crosses for seed production and six of eight crosses for viable seeds. Moreover, for both directions, the asymmetries were significant at *P* < 0.0001. Note that these data are corrected for differences in the potential of parental taxa to set seed or in the proportion of viable seeds produced under experimental conditions.

Interestingly, the direction of asymmetry at germination was reversed (Fig.1B). Germination rates of the fewer F_1_ hybrid seeds produced when *A. arenosa* was the maternal parent were significantly higher than germination rates of *A. lyrata* maternal F_1_ hybrid seeds, despite producing more seeds (Mann–Whitney *U*-test, *P* = 0.027).

### Expected development failure of low germinating *A. lyrata* maternal F_1_ seeds not apparent from PCA

**Figure 2:**
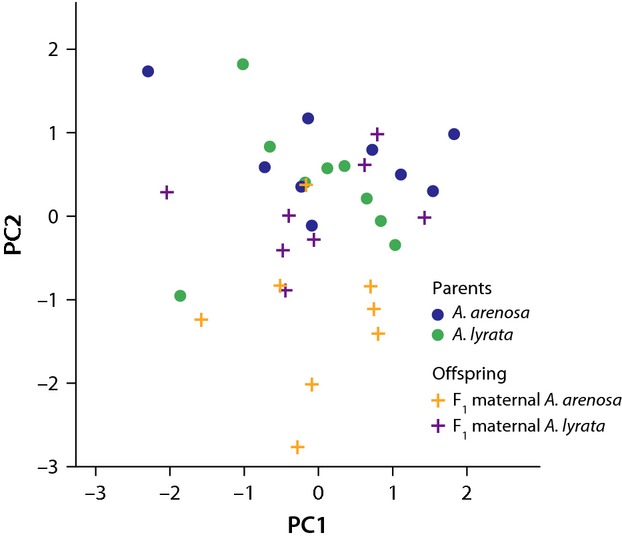
PCA projection of seed morphology (as a proxy of endosperm development) measured in *A. arenosa*, *A. lyrata*, and their F_1_ offspring. First two principal components from a PCA analysis of seed morphology measured from F_1_ seeds of conspecific crosses (*A. arenosa*, blue and *A. lyrata*, green circles, respectively) and heterospecific crosses (F_1_ maternal *A. arenosa*, orange and F_1_ maternal *A. lyrata*, purple crosses, respectively).

The low germination of *A. lyrata* maternal F_1_'s may be a result of endosperm development failure (Haig 2013), which would be evident in the size and/or appearance of nongerminating seeds. In the absence of dissecting fertilized ovules to check for incomplete development, we used a PCA of seed morphology in the parents and the F_1_, as a proxy for endosperm overgrowth (large seeds). Interestingly, the low germinating F_1_ *A. lyrata* seeds are subsumed in the same cloud as their parents, that is, the expected development failure is not apparent (Fig.2). On the other hand, the higher germinating F_1_ maternal *A. arenosa* seeds are noticeably smaller. They sit outside the main cloud containing both parents/F_1_ maternal *A. lyrata*. This suggests that development failure might not be a factor in the low germination of *A. lyrata* maternal F_1_ hybrids. However, endosperm dissection data would be required to confirm this.

An analysis of variance on germination rates (as the response variable) indicated that the source of the seed (parent as a fixed factor) was statistically significant (*P* = 0.013) while seed size (covariate) and the interaction between the two were not significant (*P* = 0.105 and *P* = 0.193, respectively), suggesting that while seed size appears to have no effect on seed viability, the maternal parent is a significant component of seed fitness in our experiment.

## Discussion

Several prezygotic mechanisms may account for the asymmetries in seed set observed in this study. Self-incompatible species may be less receptive to foreign pollen than self-compatible species, for example, leading to significant asymmetries in hybrid seed set between the two mating types (Lewis and Crowe 1958). Both species, however, are self-incompatible (SI), leaving this explanation unlikely. In addition, although detailed analysis of the maternal component of the SI system (*S* receptor kinase, SRK) and its segregation in our F_1_ families (Appendix 1, TableA1) showed some evidence for segregation distortion, this was often due to selection against homozygotes, which is expected for loci involved in self-incompatibility (due to strong inbreeding depression). Similar distortion was found for conspecific as well as heterospecific crosses and so there is no evidence that the *S* locus might be involved in selection against hybrids.

Differential fruit abortion may account for the asymmetry in seed set. Variation in reproductive success may occur because pollen competes for access to ovules or because seed parents differentially exclude pollen phenotypes (Moore and Pannell 2011). The asymmetry we observe for seed set is consistent with Kaneshiro's (1980) hypothesis of asymmetrical mate choice, predicting that pollen from an ancestral taxon (*A. arenosa*) may fertilize ovules from a derived taxon (*A. lyrata*), but not vice versa. While plants do not choose their mates in the same way female animals may actively choose (as envisaged originally by Kaneshiro 1980), discrimination among pollen grains based on the genotype expressed at SI loci, for example, is of course possible. Kaneshiro's prediction can thus be tested in plants (Tiffin et al. 2001). We observed that the isolation asymmetry between *A. arenosa* and *A. lyrata* was not (100%) complete, however, suggesting that barriers to gene flow between these two species may be reversed over the course of species divergence (e.g., Fuller 2008).

### *Arabidopsis lyrata* maternal hybrids are more successful in the wild – contra predictions based on chloroplast evolution

Asymmetries in postzygotic incompatibility between plant species are less well documented particularly the asymmetry reversals between life history stages reported here. Postzygotic isolation may result from several types of nuclear–cytoplasmic interactions (Burton et al. 2013). Cytoplasmic male sterility elements may be responsible for the asymmetric hybrid viabilities if male sterility in the maternal parent is not restored by nuclear genes in the F_1_ (hybrid) background. In reciprocal crosses between species, the maternal parent with faster cytoplasm evolution will tend to produce less viable F_1_ hybrids (lower germination rates) owing to an increased probability of cytonuclear incompatibilities (Turelli and Moyle 2007). We tested this prediction using whole chloroplast genome data and molecular evolution rates from a clade of *Arabidopsis* close relatives including *A. arenosa* and *A. lyrata* (Appendix 2; Fig.A1, TableA2). As predicted, the species which tended to be the inferior maternal parent for F_1_ hybrids (*A. lyrata*; seed viability) exhibited accelerated chloroplast genome evolution, providing comparative evidence for a systematic basis to Darwin's corollary. This result is consistent with the hypothesis that cytonuclear incompatibilities can play an important role in reproductive isolation in our reciprocal crosses. However, such asymmetrical reproductive isolation does not explain the direction of asymmetrical chloroplast introgression observed between *A. arenosa* and *A. lyrata* in natural suture zones, where *A. lyrata* tends to be the hybrid maternal parent (Schmickl and Koch 2011; Koch et al. unpubl. data). This suggests that there may be further delayed hybrid breakdown of fertility at later developmental stages or that later-acting selection against *A. arenosa* maternal hybrids (unrelated to hybrid fertility, e.g. substrate adaptation Schmickl and Koch 2011) is responsible for the apparent excess of *A. lyrata* maternal hybrids in the wild.

The majority of diploid *A. lyrata* populations in the wild grow on calcareous outcrops in the east Austrian Forealps, but populations also grow on siliceous bedrocks, for example, the Bohemian Massif in the Czech Republic (Schmickl et al. 2010), suggesting either the presence of local edaphic adaptation or extreme physiological plasticity within this species. Similarly, within diploid *A. arenosa*, calcicole populations occur exclusively in the Carpathians and the Balkan Peninsula, while siliceous populations are mainly restricted to the High Tatras (Schmickl et al. 2012). This substrate specialization has led to spatial separation of ecological populations within both species. The role of substrate adaptation, however, in shaping both this diversification and the fitness of heterospecific hybrids is unknown.

One way to test whether exogenous, rather than endogenous, selection shapes hybrid fitness would be to investigate the sensitivity of germination and early seedling growth to substrate of origin by comparing the performance of F_1_ hybrids with their parents (“home versus away” contrast sensu Kawecki and Ebert 2004). Genotype × genotype interactions (endogenous selection) should result in deviations from expectation under additive genetic architecture (Lynch and Walsh 1998). We therefore expect that if intergenomic (or cytonuclear) incompatibilities are weak (or absent), trait values for the F_1_ hybrids will equal the pooled average of the parents (Rhode and Cruzan 2005). This is indeed what we observe. Seed set and germination of artificially generated F_1_ hybrids do not exceed the worst performing parent (Fig.1).

Given that the predicted accelerated rates of chloroplast genome evolution in *A*. *lyrata* are not accompanied by an asymmetrical fitness of maternal F_1_ *A*. *lyrata* in the wild, we suggest that divergence in local substrate adaptation may be subject to parent–offspring coadaptation and that isolation barriers are likely to be environmentally dependent (exogenous) rather than endogenous.

Substrate treatments were not included in our experiment, however, and so future garden experiments will need to include heterospecific crosses (also between substrate ecotypes) to investigate how selection (exogenous vs. endogenous) could offset the decreased fitness of any new migrate allele both in a new hybrid genetic background (Dobzhansky 1937; Barton and Hewitt 1985; Barton 2001) and the substrate in which new migrant alleles are expressed (genotype × environment interactions; Barton and Gale 1993).

### Directional asymmetry at seed set is reversed in predicted direction at seed viability

The reversal in asymmetry between life stages is curious, because nuclear cytoplasmic interactions should be apparent in both seed set and their inherent viability. One could argue that as the patterns for seed set and germination are diametrically opposite, the effects cancel each other out. On the other hand, germinating at a low frequency (despite high abundance) for the long(er)-lived perennial *A. lyrata* may be a better life history strategy than germinating at high frequency to produce founding populations, as is evident for the colonizer *A. arenosa* (Donohue 2009; Rajon et al. 2009).

Seed germination rates are notoriously variable across environments even within species (for *Arabidopsis*, see Donohue et al. 2005; Montesinos-Navarro et al. 2012) and so broader population sampling is required to capture all of the variance among sites between species in this biological system. That we detected no significant difference in seed mass between the two cross types may argue against any viability interpretation based on germination. The artificial environment used in our experiment may not have been conducive to germination for *A. lyrata*, commensurate with the contradictory results from the field where *A. lyrata* maternal hybrids prevail. Finally, germination is of course a difficult fitness trait to interpret because failure to germinate may actually be the best strategy (Simons and Johnston 2006; Childs et al. 2010). Different maternal effects between the two species, whatever their ultimate basis, may not be surprising in this sense, and those effects should not necessarily go in the same direction for all traits – not least because the directionality of traits is difficult to define, particularly for germination (Donohue 2009).

## Conclusion

In *Arabidopsis* (*A. arenosa* and *A. lyrata*), the direction of isolation asymmetry between hybridizing species in the wild does not vary predictably with the relative rate of chloroplast and nuclear evolution in parental species detected here; a pattern that is not consistent with theoretical predictions (Turelli and Moyle 2007). Our data do not allow us to test whether differences in seed viability (having used a proxy), or dormancy, contribute to isolation asymmetry between these two species. If dormancy is misregulated, preventing germination, then many interesting questions regarding maternal versus embryonic control of dormancy arise (Donohue 2009) beside related issues of parent–offspring conflict (Ellner 1986) and bet-hedging (Slatkin 1974; Simons and Johnston 2006; Childs et al. 2010).

## Figures and Tables

**Figure 3:**
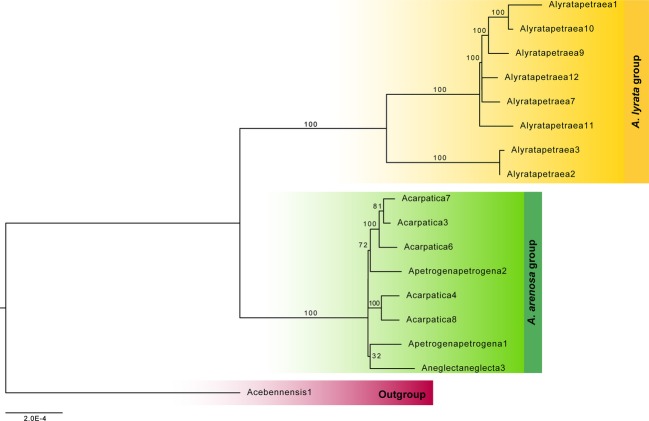
Comparison of chloroplast substitution rates between *A. arenosa* and *A. lyrata*, using *Arabidopsis cebennensis* as an outgroup. The maximum likelihood tree depicts rates of synonymous and nonsynonymous substitution based on ∼127 kbp from whole chloroplast genome sequences. Values indicate node support; bootstrap values estimated using RAxML (Stamatakis 2014). The rate differences between *A. arenosa* and *A. lyrata* for whole genome sequences were highly significant based on the relative rate test (Tajima 1993) implemented in MEGA (Tamura et al. 2013). TableA2 summarizes the results of relative rate tests performed for all pairwise heterospecific comparisons in MEGA (including both synonymous and nonsynonymous sites). All comparisons exhibited a significant difference in rates between the two species at the *P *= 0.01 threshold (without corrections for multiple testing).

## Appendix 1 Segregation distortion in SRK alleles

The following section describes the segregation of genotypes in F_1_ progeny at the *S* receptor kinase locus which controls the female component of self-incompatibility (SI).

Exchanges of *S* alleles between hybridizing species can play an important role in the evolution of *S* alleles with mutation altering pistil specificities (e.g., Chookajorn et al. 2004). To examine the asymmetries in reproductive success from the main crossing experiments in the context of the transmission of gametes, we studied segregation ratios in the progenies by formally testing whether the observed genotypic frequencies at the *SRK* locus fell outside the expected Mendelian segregation ratios for F_1_ progeny (within families) of heterospecific crosses.

### Background

Genes contributing to post pollination may play a role in prezygotic barriers. Functional products (*S* RNases) of the *S* locus, responsible for SI within species, are among the few genes that have been functionally validated as contributors to interspecific pollen rejection (Murfett et al. 1996; Li and Chetelat 2010; Tovar-Méndez et al. 2014). As such, the *S* locus has a probable role in the evolution of heterospecific incompatibility (Lewis and Crowe 1958; Hiscock and Dickinson 1993; Hancock et al. 2003).

In Brassicaceae, SI is under the genetic control of a single locus, the *S* locus, which contains two tightly linked, highly polymorphic genes, *S* receptor kinase (SRK) expressed at the (female) stigmatic surface, and its ligand *S* locus cysteine-rich protein (SCR), expressed at the (male) pollen surface. These two recognition proteins determine the SI response (Schopfer et al. 1999; Takasaki et al. 2000; Takayama et al. 2000; Silva et al. 2001). Self-pollination is prevented when the *SRK* and *SCR* of an *S* allele express the same self-incompatibility type (reviewed in Ivanov et al. 2010; Tantikanjana et al. 2010; Iwano and Takayama 2012).

A bias in the exchange of *S* alleles among closely related hybridizing species can be detected via crossing experiments and segregation analysis at the *S* locus. If an *S* allele is absent from one species, a hybrid carrier of this allele will have a strong mating advantage under balancing selection offsetting its decreased hybrid fitness and leading to selection for its introgression (Castric et al. 2008). This assumes that the *S* allele is unimpeded by linked genes maladapted in the recipient species. While the introgression of *S* alleles has the potential to be adaptive, maladapted-linked genes (negative interactions among loci or Dobzhansky–Muller incompatibilities) may lead to segregation disadvantage or fitness differences associated with SI alleles in crosses between two hybridizing species (Coyne and Orr 2004; Bomblies et al. 2007).

Segregation distortion is also possible for conspecific crosses. At the *S* locus, complementary expression of pollen and pistil specificity is essential for self-incompatibility. Crossover events permit self-fertilization by generating an *S* allele that can determine pistil rejection of a pollen specificity that differs from the one it encodes. Recombination is thus suppressed at the *S* locus but as a consequence, the locus may accumulate a genetic load within an allelic class. The latter occurs because frequency-dependent selection will shelter deleterious alleles linked to the *S* locus when their frequency gets low in the population (Uyenoyama 2005). It is therefore possible that some *S* haplotypes accumulate a more severe genetic load than others, leading to segregation distortion in crosses between *S* haplotypes within species.

### Materials and Methods

#### SRK genotyping (of parents and hybrid F_1_ generation)

Cleaved amplified polymorphism sequence (CAPS) analysis was used to determine the *S* haplotype of both parents and the progeny of each cross. Genotypes were determined by restriction digest profiles of PCR products amplified using a combination of allele-specific and degenerate primers run on high resolution polyacrylamide gels; detailed in Schierup et al. (2001); Charlesworth et al. (2000) and Mable et al. (2003). Briefly, *SRK* variants were determined by PCR-based screening using allelic-specific (forward) primers anchored in the extracellular *S* domain with the same degenerate reverse primer (SLGR), designed for diploid populations in *A. lyrata* (Schierup et al. 2001). Strong amplification products from the *S* locus were digested using the restriction endonucleases Alu I and Msp I, and the resulting CAPS fragments were visualized on a 12% polyacrylamide gel.

Diploid CAPS fragments were comparatively easy to score while tetraploid fragments were harder to determine; conspecific tetraploid crosses from *A. arenosa* subsp. *arenosa* were included as a test for functionality of self-incompatibility in this species. Given the scoring difficulties, tetraploid PCR products that generated distinctive CAPS profiles were cloned into pCR TOPO plasmids using TA Cloning (Invitrogen, Carlsbad, CA) prior to sequencing. To identify changes arising out of errors in the PCR, multiple clones for each individual were sequenced. Sequencing templates were prepared from overnight cultures using standard protocols. Clones were sequenced using DYEnamic ET Terminator Cycle Sequencing chemistry (GE Healthcare, Buckinghamshire, UK) according to the manufacturer's instructions and run on a MegaBACE 500 Sequencer. Sequences were edited, aligned, manually adjusted in SeqMan Pro (DNASTAR, Madison, WI), and submitted to BLAST. All submissions showed significant sequence homology to other *SRK* sequences in the NCBI database. Sequences have been submitted to GenBank under accession numbers (JX464611-JX464655).

#### SRK segregation analysis

To test for segregation distortion of *SRK* genotypes in the F_1_ progeny of the artificial crosses, we tested for departures from Mendelian inheritance using chi-square (χ^2^) goodness of fit tests.

Specifically, we analyzed the transmission of *S* alleles from the parental plants to the progeny surviving to the seedling stage; any deviations observed may thus be due to selection at the gametophytic or early sporophytic stage. This was assessed only for diploid crosses due to the difficulty of determining the dosage of alleles in tetraploids.

### Results

Genotyping of highly polymorphic loci under balancing selection (such as *SRK*) is notoriously difficult due to the high divergence of alleles (Mable et al. 2003). This can complicate tests for segregation, but observed (and expected) segregation under the null model of Mendelian inheritance is shown in TableA1. For the interspecific crosses (which only produced sufficient numbers of progeny for testing with *A. lyrata* as the female parent), evidence of segregation distortion was found in both families (H and L). For the H family, only a single allele was resolved for each parent, but there was an absence of “null” genotypes in the progeny, suggesting that the same allele was missing in both parents (as there is expected to be strong inbreeding depression against homozygotes at the *S* locus). There were also fewer-than-expected heterozygotes between the two resolved alleles. For family L, the *A. lyrata* parent showed resolution of only a single allele, but shared the other allele with the *A. arenosa* parent; the expected genotype classes for 25 25 homozygotes and 25 x heterozygotes were thus combined. Although the expected numbers were too low for a reliable chi-square test, there was a complete absence of heterozygotes between the 25 allele (the *A. lyrata* copy) and the 16 allele (from *A. arenosa*) and an excess of individuals showing only the 16 allele (i.e., 16 x).

**Table A1:** Segregation tests of *S* locus genotypes against the null expectation of equal probability of transmission

Family/cross	Parental genotypes	Progeny^1^					χ^2^	*P*-value
						Total		
Interspecific^2^								
* H*		*Ah18 x*	*Ah18 3*	*x x*	*x 3*			
* A. lyrata ♀ × A. arenosa ♂*	*Ah18 x ♀ *× *x 3 ♂*	9 (5.5)	4 (5.5)	0 (5.5)	9 (5.5)	22	10.36	**0.016**
* L*			*25 16*	*x 25* or *25 25*	*x 16*			
* A. lyrata ♀ *× *A. arenosa ♂*	*25 x ♀ *× *25 16 ♂*		0 (2.75)	3 (5.5)	8 (2.75)	11	13.91	**0.001**
Intraspecific								
* M*			*18a 25*	*x 18a* or *18a18a*	*x 25*			
* A. arenosa*	*18a x ♀ *× *18a 25 ♂*		3 (5)	11 (10)	6 (5)	20	0.57	0.577
	*18a 25 ♀ *× *18a x ♂*		3 (4)	8 (8)	5 (4)	16	0.50	0.779
	*Total*		6 (9)	19 (18)	11 (9)	36	1.50	0.472
* O*^3^			*25 x* or *25 25*	*16 25*	*16 x*			
* A. arenosa*	*25 16 ♀ *× *25 25 ♂*		7 (4.5)	2 (4.5)	0	9	2.78	0.100
	*25 25 ♀ *× *25 16 ♂*		3 (5)	7 (5)	0	10	1.60	0.206
	*Total*		10 (9.5)	9 (9.5)	0	19	0.05	0.819
* R*		*16 18a*	*16 16*	*8 18a*	*8 16*			
* A. arenosa*	*16 8 ♀ *× *18a 16 ♂*	6 (3.25)	0 (3.25)	4 (3.25)	3 (3.25)	13	5.77	0.123
	*18a 16 ♀ *× *16 8 ♂*	4 (2.5)	0 (2.5)	5 (2.5)	1 (2.5)	10	6.80	0.079
	*Total*	10 (5.75)	0 (5.75)	9 (5.75)	4 (5.75)	23	11.26	**0.010**
* A*			*Cg5 14*	*x Cg5* or *Cg5 Cg5*	*x 14*			
* A. lyrata*	*Cg5 x ♀ *× *Cg5 14 ♂*		1 (2.25)	4 (4.5)	4 (2.25)	9	2.11	0.348
	*Cg5 14 ♀ *× *Cg5 x ♂*		0 (1.75)	2 (3.5)	5 (1.75)	7	8.43	**0.015**
	*Total*		1 (4)	6 (8)	9 (4)	16	9.0	**0.011**

1 Observed number of F_1_ individuals within each full-sib (heterospecific)/half-sib (conspecific) cross and genotype class. Expected values are shown in parentheses. x, missing allele; Cg, *SRK* allele similar to *Capsella grandiflora*; Ah, *SRK* allele similar to *A. halleri*.

2 For each reciprocal cross, each genotype was used both as a female (♀) and a male (♂) parent. Note that one allele is frequently missing from either (or both) parent because of inconclusive genotyping.

3 Although only a single allele was resolved in one parent for this family (O), sample sizes were sufficient to obtain a robust test of whether this was due to homozygosity or the presence of a null allele. Assuming homozygosity in one parent fit the data much better (χ^2^** **= 0.05; *P* = 0.819, see also main text).

No segregation bias was found for two of the families (M and O) resulting from intraspecific crosses of *A. arenosa*. For family M, one parent showed resolution of only a single allele and the parents shared the other allele, so genotype classes were again collapsed; this model fitted the data well (*P* = 0.472 across reciprocal crosses) and reciprocal crosses showed very similar proportions of each expected genotype class. For family O, although only a single allele was resolved in one parent, sample sizes were sufficient to obtain a robust test of whether this was due to homozygosity or presence of a null allele. Considering the presence of a null allele, we would have predicted that one-fourth of the individuals should have shown amplification of only allele 16, but this was not observed in any individuals. This scenario would have resulted in significant deviation from Mendelian expectations (χ^2^ = 10.58; *P* = 0.005). On the other hand, assuming homozygosity in one parent actually fits the data much better (χ^2^ = 0.05; *P* = 0.819). The reciprocal crosses showed deviation from expectations in different directions. However, sample sizes were small and neither was significantly different from expectations.

One *A. arenosa* intraspecific family (R) and one *A. lyrata* intraspecific family (A) showed significant deviations from expectations. Family R had all alleles resolved, but showed a complete absence of the expected homozygote class (as the parents shared an allele); this resulted in a significant deviation from expectations for the combined test across reciprocal crosses (samples sizes were too low within to produce a reliable test). Family A was again missing an allele in one parent, but showed an excess of heterozygotes for this allele with one of those from the other parent (14 x) but a lower than expected frequency of the 14 allele in combination with the shared Cg5 allele (χ^2^ = 9.0, *P* = 0.011). This pattern was observed for both reciprocal crosses, but sample sizes were too low for individual tests.

## Appendix 2 Darwin's corollary to Haldane's rule

The following section describes the test of the prediction that differences in the rates of chloroplast evolution impact which species is likely to be the inferior maternal parent in reciprocal crosses (Turelli and Moyle 2007; Bolnick et al. 2008). It compares the direction of F_1_ viability asymmetry, and chloroplast substitution rates, in a clade of *Arabidopsis* relatives.

### Background

We detected multiple asymmetries for reciprocal crosses between *A. arenosa* and *A. lyrata*, a pattern referred to as Darwin's corollary to Haldane's rule (Turelli and Moyle 2007). A general pattern that emerged was that F_1_ hybrids with either *A. arenosa* or *A. lyrata* as the maternal parent were affected by asymmetries depending on the life history stage. Specifically, crosses in which *A. arenosa* was the maternal parent produced significantly fewer seeds than the reciprocal cross. However, the fertility of these fewer *A. arenosa* hybrid seeds was significantly higher compared to *A. lyrata* hybrid seeds from the reciprocal cross.

Asymmetries in reciprocal crosses might occur in F_1_ hybrids due to the effects of uniparentally inherited Dobzhansky–Muller incompatibilities, such as maternal effects and cytonuclear interactions. Turelli and Moyle (2007) suggested that differences between two species in the ratio of the rates of chloroplast to nuclear evolution between two species can result in consistent asymmetries between reciprocal crosses. If the differences in these ratios are due to a systematic bias, then the species with the higher ratio of chloroplast to nuclear evolution would be predicted to be an inferior maternal parent, as was found in a clade of centrarchid fish (Bolnick et al. 2008). We tested the rates of mutation by asking whether the rate of chloroplast evolution is higher in *A. lyrata* than that of *A. arenosa* as predicted by the lower germination rates in this species.

### Results

**Table A2:** Pairwise relative rate tests for *Arabidopsis arenosa*^1^ and *A. lyrata* chloroplast genome lineages

Sequence A	Sequence B	Identical sites in all three sequences	Divergent sites in all three sequences	Unique differences in Sequence A	Unique differences in Sequence B	Unique differences in Sequence C	χ^2^	*P*-value
*Acarpatica3*	*Alyratapetraea1*	127091	0	69	134	209	20.81	0.00001
*Acarpatica3*	*Alyratapetraea2*	127110	0	69	115	209	11.5	0.0007
*Acarpatica3*	*Alyratapetraea3*	127108	0	69	117	209	12.39	0.00043
*Acarpatica3*	*Alyratapetraea7*	127109	0	68	116	210	12.52	0.0004
*Acarpatica3*	*Alyratapetraea9*	127105	0	68	120	210	14.38	0.00015
*Acarpatica3*	*Alyratapetraea10*	127106	0	69	119	209	13.3	0.00027
*Acarpatica3*	*Alyratapetraea11*	127103	0	68	122	210	15.35	0.00009
*Acarpatica3*	*Alyratapetraea12*	127110	0	68	115	210	12.07	0.00051
*Acarpatica4*	*Alyratapetraea1*	127087	0	73	134	209	17.98	0.00002
*Acarpatica4*	*Alyratapetraea2*	127106	0	73	115	209	9.38	0.00219
*Acarpatica4*	*Alyratapetraea3*	127104	0	73	117	209	10.19	0.00141
*Acarpatica4*	*Alyratapetraea7*	127105	0	72	116	210	10.3	0.00133
*Acarpatica4*	*Alyratapetraea9*	127101	0	72	120	210	12	0.00053
*Acarpatica4*	*Alyratapetraea10*	127102	0	73	119	209	11.02	0.0009
*Acarpatica4*	*Alyratapetraea11*	127099	0	72	122	210	12.89	0.00033
*Acarpatica4*	*Alyratapetraea12*	127106	0	72	115	210	9.89	0.00166
*Acarpatica6*	*Alyratapetraea1*	127088	0	72	134	209	18.66	0.00002
*Acarpatica6*	*Alyratapetraea2*	127107	0	72	115	209	9.89	0.00166
*Acarpatica6*	*Alyratapetraea3*	127105	0	72	117	209	10.71	0.00106
*Acarpatica6*	*Alyratapetraea7*	127106	0	71	116	210	10.83	0.001
*Acarpatica6*	*Alyratapetraea9*	127102	0	71	120	210	12.57	0.00039
*Acarpatica6*	*Alyratapetraea10*	127103	0	72	119	209	11.57	0.00067
*Acarpatica6*	*Alyratapetraea11*	127100	0	71	122	210	13.48	0.00024
*Acarpatica6*	*Alyratapetraea12*	127107	0	71	115	210	10.41	0.00125
*Acarpatica7*	*Alyratapetraea1*	127091	0	69	134	209	20.81	0.00001
*Acarpatica7*	*Alyratapetraea2*	127110	0	69	115	209	11.5	0.0007
*Acarpatica7*	*Alyratapetraea3*	127108	0	69	117	209	12.39	0.00043
*Acarpatica7*	*Alyratapetraea7*	127109	0	68	116	210	12.52	0.0004
*Acarpatica7*	*Alyratapetraea9*	127105	0	68	120	210	14.38	0.00015
*Acarpatica7*	*Alyratapetraea10*	127106	0	69	119	209	13.3	0.00027
*Acarpatica7*	*Alyratapetraea11*	127103	0	68	122	210	15.35	0.00009
*Acarpatica7*	*Alyratapetraea12*	127110	0	68	115	210	12.07	0.00051
*Acarpatica8*	*Alyratapetraea1*	127087	0	73	134	209	17.98	0.00002
*Acarpatica8*	*Alyratapetraea2*	127106	0	73	115	209	9.38	0.00219
*Acarpatica8*	*Alyratapetraea3*	127104	0	73	117	209	10.19	0.00141
*Acarpatica8*	*Alyratapetraea7*	127105	0	72	116	210	10.3	0.00133
*Acarpatica8*	*Alyratapetraea9*	127101	0	72	120	210	12	0.00053
*Acarpatica8*	*Alyratapetraea10*	127102	0	73	119	209	11.02	0.0009
*Acarpatica8*	*Alyratapetraea11*	127099	0	72	122	210	12.89	0.00033
*Acarpatica8*	*Alyratapetraea12*	127106	0	72	115	210	9.89	0.00166
*Apetrogenapetrogena1*	*Alyratapetraea1*	127090	0	70	132	211	19.03	0.00001
*Apetrogenapetrogena1*	*Alyratapetraea2*	127108	0	71	114	210	9.99	0.00157
*Apetrogenapetrogena1*	*Alyratapetraea3*	127106	0	71	116	210	10.83	0.001
*Apetrogenapetrogena1*	*Alyratapetraea7*	127107	0	70	115	211	10.95	0.00094
*Apetrogenapetrogena1*	*Alyratapetraea9*	127103	0	70	119	211	12.7	0.00036
*Apetrogenapetrogena1*	*Alyratapetraea10*	127105	0	70	117	211	11.81	0.00059
*Apetrogenapetrogena1*	*Alyratapetraea11*	127101	0	70	121	211	13.62	0.00022
*Apetrogenapetrogena1*	*Alyratapetraea12*	127108	0	70	114	211	10.52	0.00118
*Apetrogenapetrogena2*	*Alyratapetraea1*	127088	1	72	133	209	18.15	0.00002
*Apetrogenapetrogena2*	*Alyratapetraea2*	127106	0	73	115	209	9.38	0.00219
*Apetrogenapetrogena2*	*Alyratapetraea3*	127104	0	73	117	209	10.19	0.00141
*Apetrogenapetrogena2*	*Alyratapetraea7*	127106	1	71	115	210	10.41	0.00125
*Apetrogenapetrogena2*	*Alyratapetraea9*	127102	1	71	119	219	12.13	0.0005
*Apetrogenapetrogena2*	*Alyratapetraea10*	127103	1	72	118	209	11.14	0.00085
*Apetrogenapetrogena2*	*Alyratapetraea11*	127100	1	71	121	210	13.02	0.00031
*Apetrogenapetrogena2*	*Alyratapetraea12*	127107	1	71	114	210	9.99	0.00157
*Aneglectaneglecta3*	*Alyratapetraea1*	127082	0	78	134	209	14.79	0.00012
*Aneglectaneglecta3*	*Alyratapetraea2*	127100	0	79	116	208	7.02	0.00806
*Aneglectaneglecta3*	*Alyratapetraea3*	127098	0	79	118	208	7.72	0.00546
*Aneglectaneglecta3*	*Alyratapetraea7*	127099	0	78	117	209	7.8	0.00522
*Aneglectaneglecta3*	*Alyratapetraea9*	127095	0	78	121	209	9.29	0.0023
*Aneglectaneglecta3*	*Alyratapetraea10*	127097	0	78	119	209	8.53	0.00349
*Aneglectaneglecta3*	*Alyratapetraea11*	127093	0	78	123	209	10.08	0.0015
*Aneglectaneglecta3*	*Alyratapetraea12*	127100	0	78	116	209	7.44	0.00637

1 Diploid representatives of the *Arabidopsis arenosa* aggregate, namely *A. carpatica*, *A. petrogena*, and *A. neglecta* (Schmickl et al. 2012). Assuming that chloroplast (cp) lineages in both species have maintained their function and are exposed to similar evolutionary constraints, then they should show similar rates of evolution. If some lineages have experienced accelerated cp evolution, then these lineages are expected to show elevated rates of evolution. To discriminate between these two hypotheses, we conducted a relative-rate test (Tajima 1993) between all pairwise heterospecific sequences (denoted as “A” and “B”) using a whole cp genome sequence from *Arabidopsis cebennensis* as an outgroup. The results consistently indicate a significantly lower substitution rate for *A. arenosa* than for *A. lyrata*. Rate constancy can thus be rejected at the 1% level for the whole cp genome between these two species.
